# Quantifying the Linguistic Complexity of Pan-Homophonic Events in Stock Market Volatility Dynamics

**DOI:** 10.3390/e28010090

**Published:** 2026-01-12

**Authors:** Yunfan Zhang, Jingqian Tian, Yutong Zou, Xu Zhang, Xiao Cai

**Affiliations:** 1 Department of FinTech, Nanjing University of Information Science and Technology, Nanjing 210044, China; 202363050018@nuist.edu.cn (Y.Z.); 202363050013@nuist.edu.cn (Y.Z.); zx8387@126.com (X.Z.); 2 Laboratory of Philosophy and Social Sciences at Universities in Jiangsu Province-Fintech and Big Data Laboratory of Southeast University, Southeast University, Nanjing 211189, China; 3 Research Center of Applied Electromagnetics, Nanjing University of Information Science and Technology, Nanjing 210044, China; caixiao@nuist.edu.cn

**Keywords:** pan-homophonic events, spillover effects, linguistic amplitude segmentation, DCC-GARCH model, RSR model

## Abstract

Pan-Homophonic events denote fluctuations in stock prices that are triggered by phonetic similarities between event keywords and stock tickers. As a relatively novel and under-researched phenomenon, they mirror a subtle yet influential behavioral deviation within financial markets. Centering on the case of Chuandazhisheng, this study delves into how such events produce dynamic and time-varying impacts on stock prices. A linguistic amplitude segmentation method is devised to discriminate between high- and low-intensity events based on information entropy. To separate pan-homophonic-driven price movements from broader market trends, the Relational Stock Ranking (RSR) model is integrated with a Dynamic Conditional Correlation-Generalized Autoregressive Conditional Heteroskedasticity (DCC-GARCH) framework to establish an adjusted price benchmark. The empirical analysis reveals a sequential price response: initial moderate fluctuations in the low-amplitude phase often yield to more prominent volatility in the high-amplitude phase. While price surges typically occur within one or two days of the event, they generally revert within approximately three weeks. Moreover, repeated exposures to homo- phonic stimuli seem to attenuate the response, indicating a decaying spillover pattern. These findings contribute to a more profound understanding of the intersection between linguistic cues and market behavior and provide practical insights for investor education, information filtering, and regulatory supervision.

## 1. Introduction

Over the past decade, China’s stock market has exhibited frequent anomalies such as the Calendar Effect, Momentum Effect, and Reversal Effect. These anomalies challenge the Efficient Market Hypothesis (EMH) [1] by revealing deviations between market prices and intrinsic values, as well as irrational components in investors’ behavior [2,3,4]. These deviations echo Shiller’s critique of excessive volatility [5]. A particularly intriguing phenomenon is the Pan-Homophonic Effect, where stock prices fluctuate due to events whose names or keywords are phonetically similar to stock symbols. For instance, after Gu Ailing won the gold medal at the Winter Olympics in 2022, the stock of Yuanwanggu surged twice consecutively. Similarly, during the 2023 Lunar New Year, the A-share listed company Tubaobao saw its stock price soar for two consecutive days. These instances underscore the significant impact of the Pan-Homophonic Effect on China’s stock market, which align with underreaction–overreaction models [6].

Despite its evident influence, the Pan-Homophonic Effect remains underexplored in academic literature. Existing studies primarily focus on short-term market reactions without delving into the long-term implications of such pan-homophonic events on stock price dynamics. Moreover, there is a lack of comprehensive frameworks to analyze the amplitude and intensity of these events, limiting our understanding of how different types of pan-homophonic events influence market behavior.

This study aims to address these gaps by introducing a novel approach that applies linguistic amplitude segmentation to categorize pan-homophonic events into high- and low-amplitude types based on their deviation magnitude. By combining the Stock Relationship Network Prediction (RSR) model with the Dynamic Conditional Correlation-Generalized Autoregressive Conditional Heteroskedasticity (DCC-GARCH) model, we establish a baseline stock price unaffected by pan-homophonic effects, allowing for a more nuanced analysis of both short-term and long-term impacts. This framework not only provides a comprehensive analysis of the amplitude and intensity of pan-homophonic events but also offers new insights into the spillover effects and the evolutionary mechanisms of stock price anomalies under the Pan-Homophonic Effect.

This study contributes to the literature in two principal aspects. First, it integrates linguistic amplitude segmentation with advanced machine learning models to analyze the Pan-Homophonic Effect. By categorizing events based on their amplitude, we can better understand the varying impacts of different types of homophonic events on stock prices. Second, it employs the RSR and DCC-GARCH models enables a dynamic and comprehensive examination of stock price movements, providing valuable insights into the mechanisms underlying market anomalies.

## 2. Literature Review

### 2.1. Pan-Homophonic Effect Research

**Figure 1:**
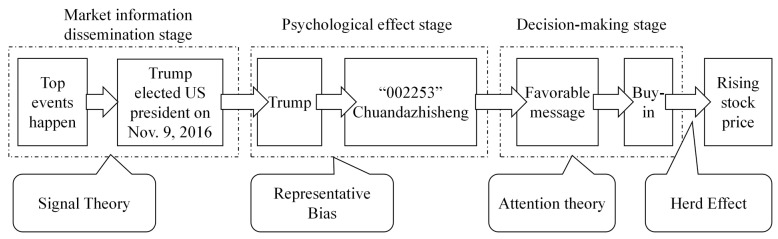
Theoretical Analysis of the Pan-Harmonic Effect.

Previous research has explored the role of Pan-Homophonic Effects [7] in financial markets, particularly in China, from various dimensions. Du and Liu highlighted that pan-homophonic events in the Chinese stock market can trigger short-term price anomalies through investors’ psychological mechanisms. The core logic is that investors overreact to phonetic associations—media amplifies the significance of these events, leading investors to mistakenly attribute stock price movements to non-fundamental factors, ultimately causing market volatility. As illustrated in Figure 1, this process is directly related to selection bias [8] and herd behavior [9] in behavioral finance: investors tend to focus on pan-homophonic cues, and follow herd behavior, trading based on group decisions, which drives up stock prices in the short term. However, existing research has not developed a quantitative framework for analyzing the intensity of the effects of pan-homophonic events. For instance, there is significant variation in the degree of stock price deviation caused by pan-homophonic effects at different stages, yet the academic community lacks a systematic classification of high-amplitude resonance period and low-amplitude resonance period. This study introduces the Linguistic Amplitude Segmentation method to fill this gap, quantifying event types based on the magnitude of stock price deviation. This method addresses the impact intensity dimension that Du and Liu did not cover and provides a new perspective for analyzing differential market responses.

### 2.2. Behavioral Foundations of the Pan-Homophonic Effect

From a behavioral finance perspective, the pan-homophonic effect can be understood as a composite mechanism driven by salience-induced limited attention, associative activation in belief formation, and noise-trader sentiment. Rather than reflecting new fundamental information, pan-homophonic cues operate as non-informational linguistic signals that become salient under specific market and media environments, thereby influencing investor behavior through cognitive rather than rational channels.

First, the effect is closely related to the literature on limited attention and attention-driven trading. Investors face severe information constraints and tend to allocate attention selectively toward salient and easily processed cues, particularly during periods of heightened market activity or media exposure. Seminal studies show that attention-grabbing events can systematically bias trading behavior toward highlighted assets, even in the absence of fundamental news [10,11]. In the context of pan-homophonic events, phonetic similarity amplifies salience by lowering cognitive processing costs, making the associated stocks more likely to attract attention-driven buying pressure. Importantly, unlike ordinary news-driven trading where the news carries substantive links to the firm, pan-homophonic effects occur even when the media event has no fundamental relevance to the stock, affecting investor behavior solely via linguistic resonance.

Second, pan-homophonic effects are consistent with associative-activation mechanisms emphasized in behavioral models of belief formation. Psychological evidence suggests that investors rely on heuristic-based associations, such as availability and representativeness, when processing complex or ambiguous information [12]. Asset-pricing models incorporating biased belief updating demonstrate that salient but irrelevant cues may induce temporary overreaction followed by correction [13]. Linguistic resemblance serves as a trigger for such associative activation, causing investors to overweight coincidental phonetic links when forming expectations about price movements.

Third, the pan-homophonic effect aligns with the noise-trader sentiment framework, in which non-fundamental signals generate correlated trading behavior and excess volatility [14]. Because phonetic cues carry no intrinsic valuation information, their impact is inherently transient and prone to reversal once attention dissipates or sentiment normalizes. This framework also provides a theoretical basis for why pan-homophonic effects are short-lived, attenuate with repeated exposure, and are more pronounced among retail investors who are more susceptible to sentiment and attention shocks.

Importantly, existing research on linguistic and name-based effects in finance further supports this interpretation. Studies on processing fluency and name pronounceability show that linguistically fluent or familiar cues can influence judgment and perceived attractiveness, even in financial decision-making contexts [15]. Related evidence from firm name changes, ticker-symbol effects, and rebranding episodes indicates that linguistic features can temporarily affect investor perception and trading behavior without altering fundamentals.

Thus, the pan-homophonic effect represents a salience-driven, linguistically mediated attention shock that propagates through investor networks and dissipates over time.

### 2.3. Spillover Effects of Extreme Events on Stock Prices

Behavioral finance research on the spillover effects of extreme events has revealed a deep connection between investor attention and market volatility. Related evidence indicates that under stress conditions induced by extreme events, economic agents tend to overweight salient cues and exhibit loss-averse behavior, thereby deviating systematically from rational benchmarks [16]. Attention Theory [17] suggests that media coverage of hot events significantly increases attention to related stocks, triggering investors’ limited attention bias—meaning investors are more likely to trade the stocks featured in the media rather than base their decisions on fundamental analysis. Media-driven sentiment effects are consistent with Tetlock [18]. This attention-driven trading behavior [18], combined with herd behavior, leads to sharp short-term fluctuations in stock prices, followed by a correction phase, confirming the classic pattern of short-term overreaction—long-term mean reversion [19]. Evidence from Chinese market attention patterns also supports this mechanism [20]. It is worth noting that the Attention Spillover Effect [19] provides an explanatory framework for the long-term impact of the Pan-Homophonic Effect. This theory posits that investors’ attention to one hot stock spills over to adjacent stocks, creating correlated price movements. In the context of the Pan-Homophonic Effect, this spillover manifests as: the first pan-homophonic event leads to a sharp rise in the target stock, followed by a secondary pan-homophonic event where investors’ attention wanes, leading to weaker price surges in subsequent events—indicating a diminishing spillover effect. This mechanism aligns with the fatigue effect in herd behavior [9]—market responses to similar irrational signals diminish over time. A limitation of existing research is that most studies focus on the short-term impact of single events [10] and lack an analysis of the dynamic evolution of multi-round pan-homophonic event spillover effects. This study, by introducing the DCC-GARCH model [21,22] to quantify dynamic correlations and using the RSR [23] network model to depict stock associations, provides a systematic measurement of the cross-period spillover strength of the Pan-Homophonic Effect for the first time, filling the theoretical gap of event type differentiation on spillover effects.

## 3. Materials and Methods

### 3.1. Research Design

#### 3.1.1. Research Hypothesis

To clarify the potential transmission mechanism of the Pan-Homophonic Effect, this study proposes a theoretical hypothesis regarding its dynamic influence on stock price co-movements. We posit that this process delineates three stages: Low-Amplitude Resonance Phase, High-Amplitude Surge Phase, and High-Amplitude Drop Phase. The proposed hypothesis is as follows:

**Hypothesis 1 (H1).** 
*The Pan-Homophonic Effect induces a dynamic resonance process in stock prices which can be divided into:*

*(i)*  *Low-Amplitude Resonance Phase, characterized by mild and persistent deviations;*
*(ii)*  *High-Amplitude Surge Phase, characterized by sharp increases;*
*(iii)*  *High-Amplitude Drop Phase, characterized by rapid declines following upward bursts.*

**Figure 2:**
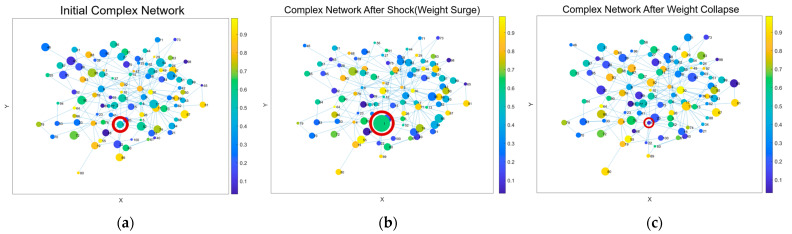
(**a**) Low-Amplitude Resonance Phase; (**b**) High-Amplitude Surge Phase; (**c**) High-Amplitude Drop Phase. Node size reflects the amplitude of stock price movements, with larger circles indicating greater price variation. Red-circled nodes denote stocks affected by the Pan-Homophonic Effect.

To facilitate intuitive understanding, a conceptual visualization is provided to illustrate the hypothesized evolution of network structures across these three phases, as shown in Figure 2, which depicts (a) the Low-Amplitude Resonance Phase, (b) the High-Amplitude Surge Phase, and (c) the High-Amplitude Drop Phase.

#### 3.1.2. Research Process

**Figure 3:**
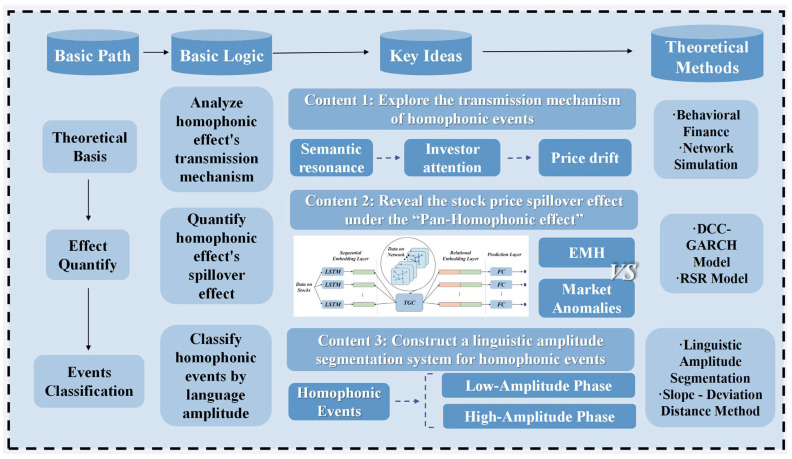
Research Framework.

The research, following the event study methodology [24,25], begins with identifying the phenomenon of the generalized pan-homophonic effect, where stock prices react to events associated with homophonic similarities between stock names or ticker symbols and external events. The overall research framework is illustrated in Figure 3. 

The first step in the research process is to establish a theoretical framework based on the EMH and Behavioral Finance, which provide the foundation for understanding the role of irrational investor behavior and attention bias in stock price movements.

Next, a complex network model is built to capture dynamic correlations between stock prices within the software and information technology services industry. This model incorporates the DCC-GARCH methodology [21,22] to assess the conditional volatility and correlations among stocks [26], allowing for a comprehensive understanding of the interactions between stocks during pan-homophonic events.

To estimate conditional variances for each stock, a univariate GARCH(1,1) [27] specification is first applied to the standardized returns $r_{t}$:

(1) $$r_{t}=\sigma_{t}\varepsilon_{t},\;\varepsilon_{t}\sim i.i.d.(0,1),$$

with the conditional variance at time t:

(2) $$\sigma_{t}^{2}=\omega +\alpha r_{t-1}^{2}+\beta \sigma_{t-1}^{2}.$$

Here, ω is the constant component of volatility, α represents the short-run reaction of volatility to shocks, β captures the persistence of volatility over time and $\varepsilon_{t}$ is the standardized innovation term.

Following this stage, standardized residuals are used to construct dynamic correlations using the DCC model [21]. The DCC recursion is defined as:

(3) $$R_{t}=diag{\left(Q_{t}\right)}^{-1/2}Q_{t}diag{\left(Q_{t}\right)}^{-1/2},$$

where

(4) $$Q_{t}=(1-a-b)\bar{Q}+a\;\varepsilon_{t-1}\varepsilon_{t-1}^{\prime }+bQ_{t-1}.$$

Here, $\bar{Q}$ denotes the unconditional covariance matrix of standardized residuals, a represents the short-term response of correlations to new shocks, b captures the persistence of correlation dynamics, $Q_{t}$ is the evolving covariance matrix before normalization and $R_{t}$ is the time-varying correlation matrix used as the dynamic graph in the proposed framework.

Following this, to establish a counterfactual benchmark that isolates stock price movements unrelated to homophonic shocks, this study adapts the original Relational Stock Ranking (RSR) model proposed by Feng et al. [23]. The RSR framework is a learning-to-rank architecture designed to jointly extract sequential and structural dependencies in financial time series. At its core, the model integrates two complementary embedding mechanisms. The temporal component employs a Long Short-Term Memory (LSTM) network to encode historical return sequences into temporal embeddings that represent the dynamic evolution patterns of individual stocks [28,29]. Recent machine-learning evidence supports such nonlinear prediction frameworks [30]. The LSTM transformation applied to a sequence $x_{t}$ is written as:

(5) $$h_{t}=LSTM(x_{t}).$$

Here, $x_{t}$ denotes the input return sequence for stock i and $h_{t}$ represents the resulting temporal embedding at time t.

Concurrently, the structural component relies on Temporal Graph Convolution (TGC) [31], which aggregates multi-relational information from a static tensor-based stock relationship graph. In the original model, the graph convolution uses:

(6) $$z_{i}=\sum_{k}A^{\left(k\right)}h_{i}W^{\left(k\right)},$$

where $A^{\left(k\right)}$ denotes the k-th relational adjacency matrix, $h_{i}$ represents the temporal embedding of stock i and $W^{\left(k\right)}$ is the learnable weight matrix associated with relation type k. This graph, constructed from industry categories and Wikipedia-derived cross-firm relations, remains fixed throughout the training process. By combining LSTM-based temporal embeddings with TGC-based relational embeddings, the RSR model effectively captures both intra-series dynamics and inter-stock structural dependencies, enabling the prediction of next-day relative return rankings under normal market conditions [32,33].

However, the original RSR formulation presumes that the inter-stock relations are static, discrete, and predefined, an assumption that is inconsistent with the time-varying nature of financial markets. The sector–industry graph and the Wikipedia-based relational tensor used in the original model remain unchanged over time; thus, the TGC model [31] can only propagate fixed structural information. In contrast, correlations among stock returns fluctuate substantially in response to market conditions, macroeconomic signals, and short-lived behavioral anomalies such as homophonic events. Capturing this dynamic co-movement is essential for constructing an accurate counterfactual benchmark. To address this limitation, the present study replaces the static graph in the RSR framework with a dynamic correlation matrix estimated using the DCC-GARCH model [21,22].

Building on these considerations, we propose an enhanced DCC-RSR model that integrates dynamic correlation structures into the relational learning component of the original RSR architecture. As summarized in Table 1, the key innovation lies in replacing the static multi-relational tensor A with a time-varying DCC-based correlation matrix $C_{t}$, which is updated on a daily basis using stock return data. The modified structural propagation becomes:

(7) $$z_{i}(t)=C_{t}h_{i}(t),$$

where $C_{t}$ denotes the DCC-based correlation matrix at time t, $h_{i}(t)$ represents the temporal embedding for stock i and $z_{i}(t)$ is the dynamically updated relational embedding.

This modification transforms the graph convolution process into a dynamic structural learning mechanism, enabling the model to propagate continuously evolving inter-stock relations through the TGC layers. The remaining components of the RSR framework, including LSTM-based temporal embedding, embedding interaction functions, and ranking-based prediction objectives, remain unchanged to ensure comparability across model variants.

**Figure 4:**
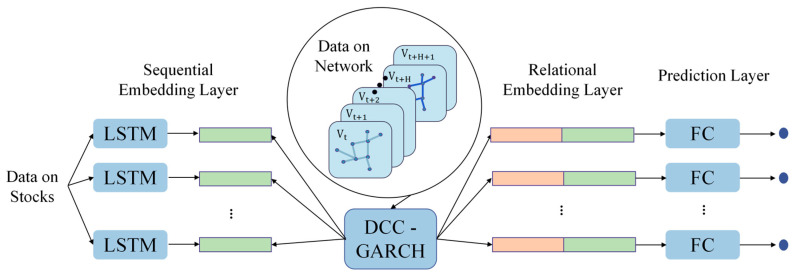
Logic Diagram of the DCC-RSR Model.

This dynamic extension allows the DCC-RSR to generate a counterfactual return ranking that reflects how the stock would behave under normal market co-movements, thereby isolating abnormal deviations induced by homophonic spillovers. The overall operational logic and data flow of the proposed model are illustrated in Figure 4, which depicts the integration of DCC-GARCH correlation estimation with sequential embedding learning and dynamic graph convolution. This enhanced architecture broadens the applicability of RSR in behavioral-finance contexts, particularly where short-lived, event-driven, or phonetic-stimulus-induced anomalies disrupt conventional price dynamics.

By comparing the predicted values with actual stock prices, abnormal deviations caused by pan-homophonic events can be identified. The analysis is further refined by categorizing pan-homophonic phases into low-amplitude and high-amplitude types based on the intensity of stock price deviations, and analyzing these stages across short-term and long-term periods to assess the evolving impact of pan-homophonic effects. This approach aligns with wavelet-based filtering techniques [34], which also highlight multi-scale market responsiveness.

Finally, a linguistic amplitude segmentation approach is employed to quantify the deviation structure and to identify the market responses associated with pan-homophonic events. The present study adopts an entropy-based deviation measure to capture the distributional complexity embedded in the prediction errors, enabling a richer characterization of the uncertainty structure underlying the observed price dynamics.

To formalize this method, let ${\hat{y}}_{t}$ denote the predicted price at time t, and $y_{t}$ the corresponding true price. Their deviation is defined as the difference variable $e_{t}={\hat{y}}_{t}-y_{t}$. Building on the seminal work of Rényi [35], the information entropy of this deviation series, representing the uncertainty and dispersion of prediction errors, is computed as:

(8) $$H=-\sum_{i=1}^{N}p_{i}\log p_{i},$$

where $p_{i}$ denotes the empirical probability of deviation values falling into the i-th bin of a discretized distribution, and N is the total number of bins used for approximation. A higher entropy value reflects a broader and more irregular dispersion of deviations, indicating greater instability in the price formation process.

To isolate the entropy component generated by pan-homophonic events, the analysis identifies non-event periods to estimate baseline uncertainty, which is then removed from the entropy observed during event periods. Let $H_{pan}$ represent the entropy of deviations during the stage potentially influenced by pan-homophonic effects, and $H_{baseline}$ represent the entropy under normal, unaffected conditions. Following Brouty and Garcin [36], the incremental entropy attributable to the pan-homophonic shock is defined as:

(9) $$\Delta H=H_{pan}-H_{baseline}.$$

with ΔH>0 indicating that the phonetic stimulus increases the entropy of deviations beyond baseline market variation, the value of ΔH is subsequently used to implement the linguistic amplitude segmentation approach. The entropy differentials are classified into three levels: no widespread harmonic effect, low amplitude resonance, and high amplitude resonance, which provides a structured basis for distinguishing the intensity of phonetic-induced deviations across periods.

To obtain an objective and statistically grounded criterion for amplitude segmentation, the threshold separating low- and high-amplitude resonance is determined using a bootstrap-based inference procedure. Specifically, the incremental entropy measure ΔH is evaluated over a reference interval that encompasses both resonance phases, and repeated resampling is employed to approximate its sampling distribution. By drawing bootstrap replicates from the deviation series and recomputing ΔH for each replicate, an empirical distribution of entropy increments is obtained, which allows for direct quantification of estimation uncertainty. The resulting bootstrap mean and confidence bounds are then used to define the entropy threshold that distinguishes low- from high-amplitude resonance in a data-driven and statistically robust manner.

#### 3.1.3. Case Selection

The selection of Chuandazhisheng as the primary case for analyzing pan-homophonic effect is based on several compelling reasons. First, it features a strong homophonic association and typical phenomenon: The name Chuandazhisheng has a notable homophonic connection to Trump, where Chuan in Chuandazhisheng sounds identical to Chuan in Trump, and Zhisheng aligns semantically with Victory. This close phonetic and semantic link to the high-profile event of Donald Trump’s U.S. presidential election significantly triggers pan-homophonic effect, leading to clear stock price anomalies that are ideal for observation and quantification.

Second, the company has experienced two significant episodes of the Pan-Homophonic Effect, making it an ideal candidate for investigating both short-term and long-term impacts. The first occurrence in 2016 saw an abnormal stock price surge driven by the homophonic association, enabling analysis of immediate market reactions and subsequent price corrections. The second occurrence in 2024 allows for comparative analysis to explore how market responses evolve over time, particularly amid increasing market maturity and investor education.

Additionally, Chuandazhisheng is representative of the software and information technology service sector, which attracts heightened media attention and investor sentiment shifts. This sector provides a rich environment for studying attention-driven market anomalies and their interaction with stock price behavior. Comprehensive historical data on stock price trends and external events further enhance the case’s suitability, enabling the research to effectively isolate pan-homophonic effect and shed light on investor behavior and market dynamics under irrational influences.

#### 3.1.4. Data Collection and Preprocessing

In this paper, the closing prices of 158 stocks in the software and information technology service industry from 2012 to 2017 were obtained from the CSMAR Guotai’an database (stocks listed after 2012 were excluded). Among them, the securities code of Chuandazhisheng is 002253. It can be observed that on 9 November 2016, the stock price increased significantly—the stock price once approached the daily limit, and the closing increase was 6.35%, achieving a significant growth in the company’s market value.

During data preprocessing, the Arima interpolation method was adopted to fill in the missing values, and the data was segmented into time series. For Chuandazhisheng, the period from 1 January 2012, to 31 December 2014, was used as the training set, the period from 1 January 2015, to 9 August 2016, was used as the test set, and the period from 10 August 2016, to 31 December 2017, was used as the control data for the prediction results.

### 3.2. Model Construction

#### 3.2.1. Construction of the Complex Network

In this study, a complex industry relationship network—specifically for the software and information technology services sector—was constructed. The DCC-GARCH model was employed to capture the dynamic correlations among stock prices. This model effectively reflects the volatility of stock prices under different market conditions by estimating conditional variance and covariance matrices. Specifically, the parameter settings of the DCC-GARCH model consist of two main components: the GARCH component, which captures the conditional volatility of individual stocks, and the DCC component, which captures the dynamic correlations among different stocks.

To avoid potential circularity between model estimation and event identification, the DCC-GARCH model is estimated solely on pre-event data. The estimation sample consists of a training period from 2012 to 2014 and a testing period from 2015 up to the event date in 2016, with no observations from the event or post-event windows used in estimating conditional variances or correlations. Based on this pre-event sample, time-varying correlation matrices are obtained and averaged over time to construct a fixed inter-stock relationship matrix. This matrix is then treated as an exogenous network input in the subsequent prediction stage, where event-period prices are used exclusively for out-of-sample evaluation.

**Figure 5:**
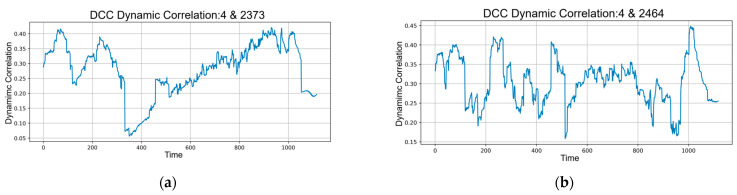

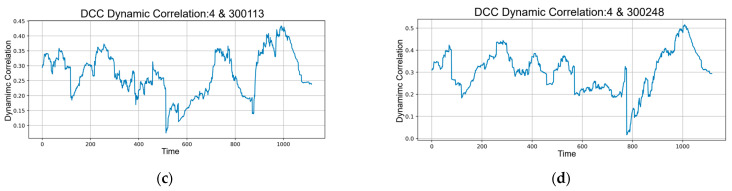
(**a**) Time-series graph of the dynamic correlation between stocks with securities codes 000004 and 002373; (**b**) Time-series graph of the dynamic correlation between stocks with securities codes 000004 and 002464; (**c**) Time-series graph of the dynamic correlation between stocks with securities codes 000004 and 300113; (**d**) Time-series graph of the dynamic correlation between stocks with securities codes 000004 and 300248.

To more rigorously illustrate the heterogeneous correlation structures identified by the DCC-GARCH framework, four representative stock pairs were selected according to the distinct behavioral patterns reflected in their dynamic co-movements, as illustrated in Figure 5.

The pair formed by stocks 000004 and 002373 demonstrates a pronounced breakdown in correlation, providing a clear example of the model’s ability to detect abrupt structural discontinuities induced by market disturbances. In contrast, the pair comprising 000004 and 002464 is characterized by persistent high-frequency oscillations with rapid directional reversals, underscoring the sensitivity of the model to short-lived noise components and frequent shifts in market sentiment. The combination of 000004 and 300113 exhibits a gradual and sustained restoration of correlation, thereby capturing the model’s capability to trace medium-term sentiment normalization processes. Finally, the pair involving 000004 and 300248 presents an uncommon transition from near-zero correlation to a strong upward rebound, ultimately attaining the highest correlation level among the selected cases; this pattern highlights the model’s capacity to accommodate both extremely low-association states and subsequent high-association regimes within a unified dynamic structure.

#### 3.2.2. Model Training and Validation

**Figure 6:**
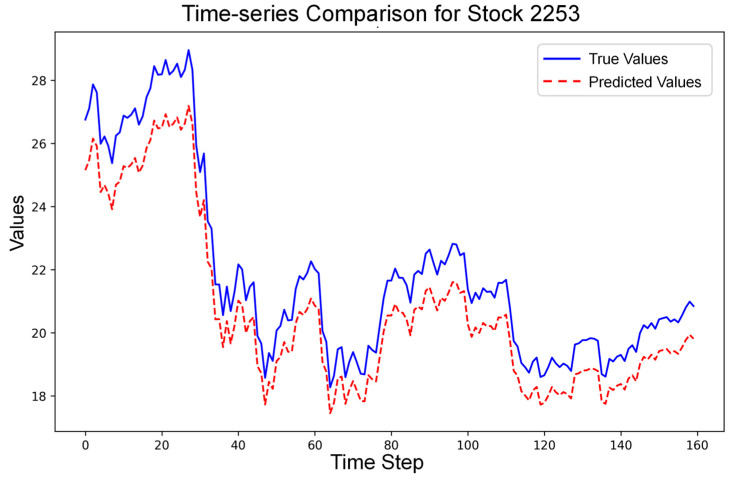
Fitting Diagram of Stock Prices in Model Prediction Results.

By inputting the stock data including the training set and the test set, as well as the dynamic correlation matrix of stocks into the Relational Stock Ranking (RSR) Model, the model successfully generates the relational rankings of 158 stocks in the industry. In the model configuration, the number of LSTM units is set to 256, and a training process of 100 rounds is arranged. The model is fully trained using the training set data. Subsequently, we introduce the test—set data for model validation. The results show that the Mean Squared Error (MSE) is approximately 1.1618, indicating that the model has good prediction performance. A specific overview of the test results is as follows:

Figure 6 presents a time-series comparison of the true stock values (in blue) and the predicted stock values (in red) for Stock 2253. The close tracking of the red dotted line (predicted values) with the blue solid line (true values) over time further validates the model’s effectiveness. Although there are some minor deviations, the overall trend is accurately captured.

**Figure 7:**
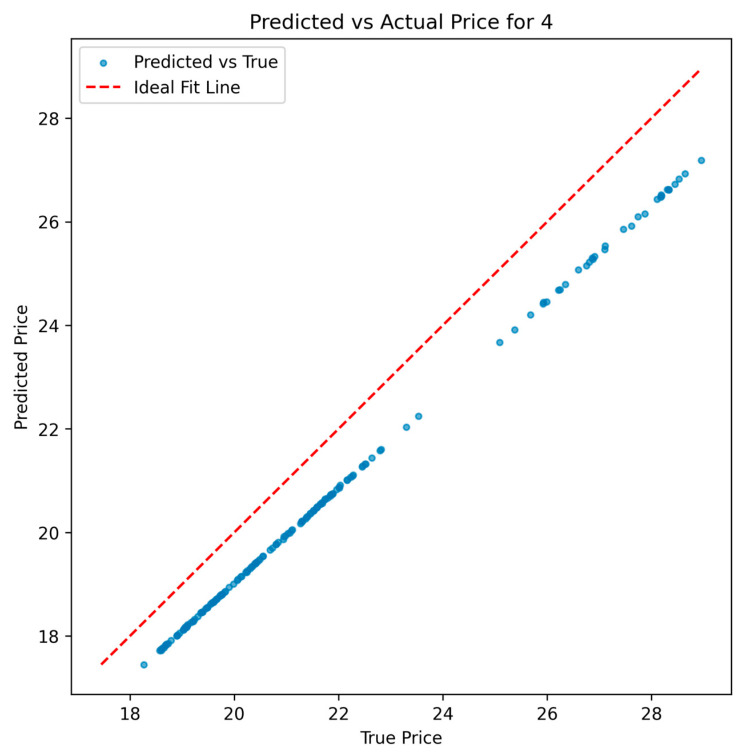
Fitting Line Chart of Model Prediction Results.

Although Figure 7 shows that the model’s predicted values tend to be slightly underestimated, it still effectively captures the overall trend of stock prices. The figure illustrates the relationship between the predicted and actual values, with data points slightly below the Ideal Fit Line, which represents the ideal scenario of perfect prediction. Nevertheless, the observed linear alignment indicates that the model adequately reflects the temporal evolution of stock prices in the market.

## 4. Results

### 4.1. Deviation of Stock Price Abnormal Movements Under the Pan-Homophonic Effect

After being trained and validated exclusively on stock price data from non-pan-homophonic periods, the Relational Stock Ranking Model is subsequently applied to predict price dynamics during time windows potentially affected by the pan-homophonic effect, as well as in the post-event period. This out-of-sample prediction design allows the model to generate benchmark price trajectories that reflect normal market co-movement patterns learned from unaffected periods, without incorporating any information from the event itself.

**Figure 8:**
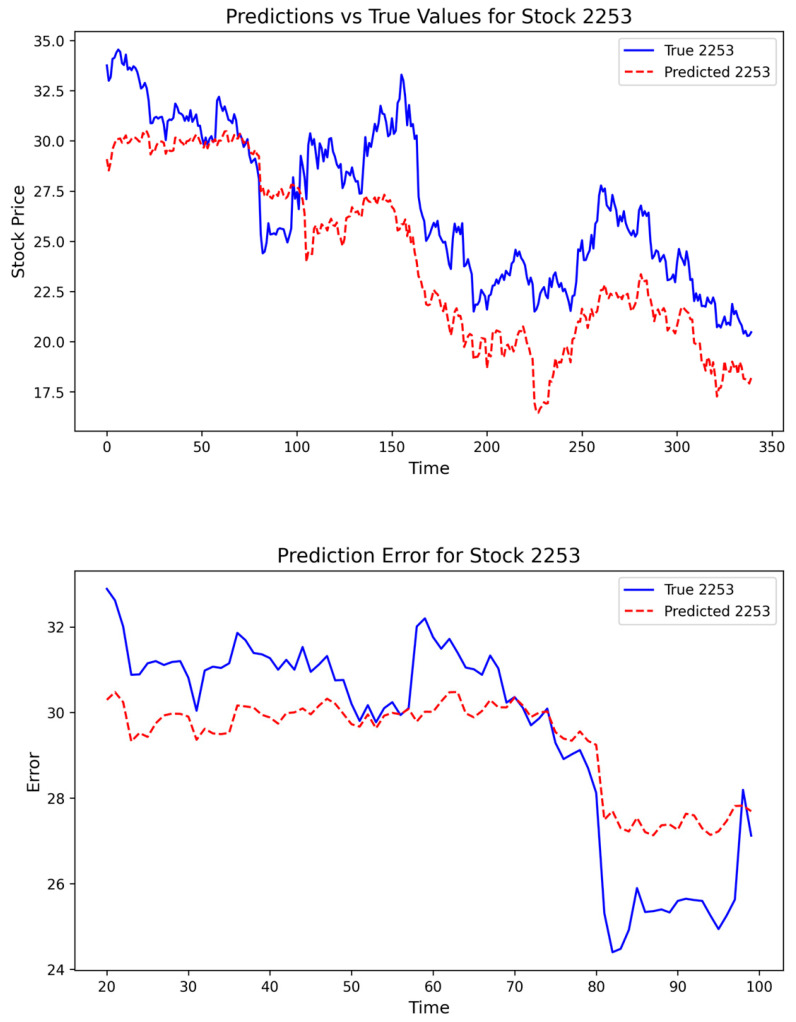
Abnormal Deviations of Stock Prices under the Pan-Homophonic Effect.

Specifically, using the model parameters estimated from the non-pan-homophonic training and testing samples, we forecast stock prices over the interval from 10 August 2016, to 29 December 2017, which covers both the pan-homophonic event window and the subsequent post-event phase. By comparing the predicted benchmark trajectories with the realized stock price movements, we assess whether and how actual prices deviate from their expected paths during periods influenced by pan-homophonic cues, as illustrated in Figure 8.

Through comparison, it can be seen that during the period when the pan-homophonic effect occurs, the stock price trend under normal conditions presents a stable and continuous state. In contrast, the pan-homophonic effect gives rise to a completely different market phenomenon. In the initial stage of its occurrence, the stock price experiences a slight deviation lasting approximately two months. Entering the outbreak stage, the stock price undergoes severe fluctuations within a short period of 1–2 days. Specifically, this fluctuation is manifested as an abnormal sharp increase in the stock price, followed by a rapid decline to below the normal fluctuation level. Moreover, the RSR is unable to predict this sharp increase. From the perspective of the long-term future trend, the pan-homophonic effect does not persist, and it disappears after approximately three weeks.

### 4.2. Linguistic Amplitude Division Based on Information Entropy

The analysis centers on an 80-day window surrounding the 2016 U.S. presidential election, during which pronounced deviations appeared in the stock’s price dynamics. The election-day announcement constitutes a natural structural break: the stock exhibited a sharp upward jump on that date, marking the transition from the low-amplitude stage to the high-amplitude stage of the pan-homophonic effect.

**Figure 9:**
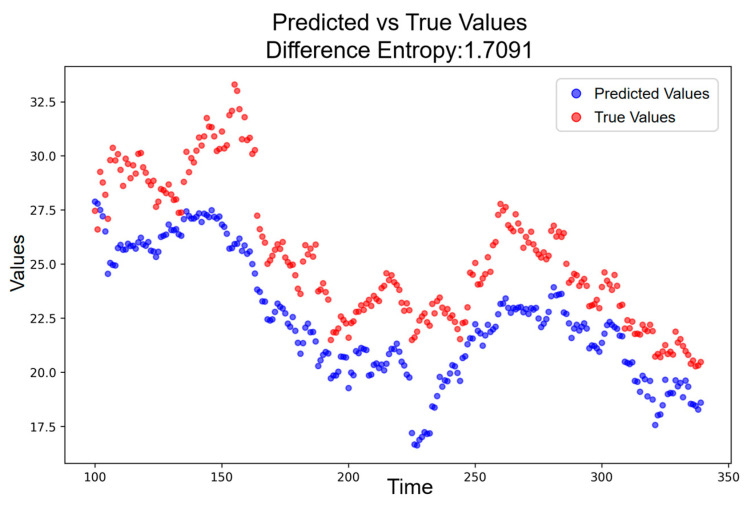
Predicted vs. Actual Price Trajectory without Pan-Homophonic Effect.

To interpret the deviation structure during the event period, it is first necessary to establish a baseline entropy level under conditions in which the pan-homophonic effect has dissipated. Accordingly, a post-event window extending from the end of the 80-day period to 31 December 2017 is selected. Within this interval, the deviation series remains stable and unaffected by phonetic stimuli. Figure 9 reports the entropy of this baseline period, which is measured as H = 1.7091. This value reflects normal market uncertainty and serves as the reference point against which event-period deviations are evaluated.

**Figure 10:**
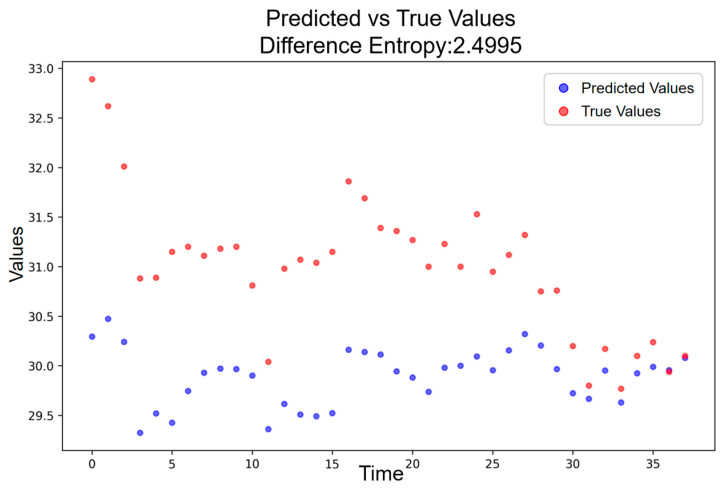
Predicted vs. Actual Price Trajectory during the Low-Amplitude Resonance Period.

During the low-amplitude resonance period, which corresponds to days prior to the announcement of the election result, the deviation structure becomes more irregular relative to the baseline. Figure 10 shows that the entropy in this phase reaches H = 2.4995, yielding an incremental phonetic-induced component of ΔH = 0.7904. This indicates that, even before the formal release of the election outcome, the phonetic resonance associated with the candidate’s name already generated a substantial increase in deviation uncertainty compared with normal conditions.

**Figure 11:**
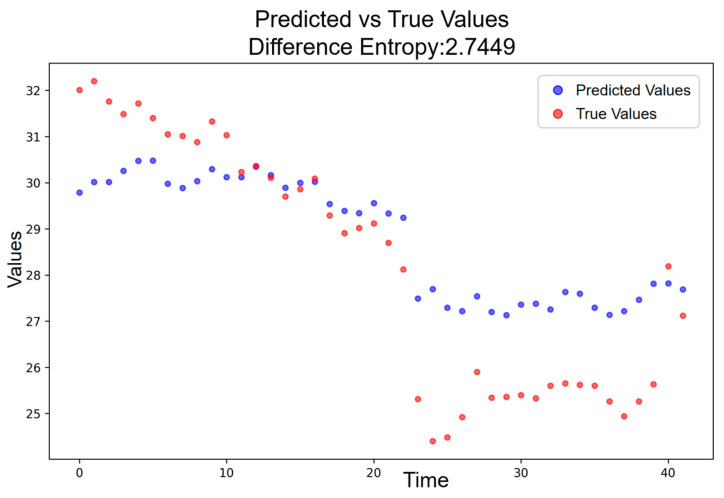
Predicted vs. Actual Price Trajectory during the High-Amplitude Resonance Period.

Following the election-day announcement, the deviation structure transitions into the high-amplitude resonance period. As shown in Figure 11, the entropy for this stage rises further to H = 2.7449, corresponding to ΔH = 1.0358. This increase is not only markedly higher than the baseline but also exceeds the ΔH observed in the low-amplitude period. The high-amplitude stage thus reflects an intensified phonetic disturbance, generating sharper irregularities and heightened uncertainty relative to the pre-announcement phase.

**Figure 12:**
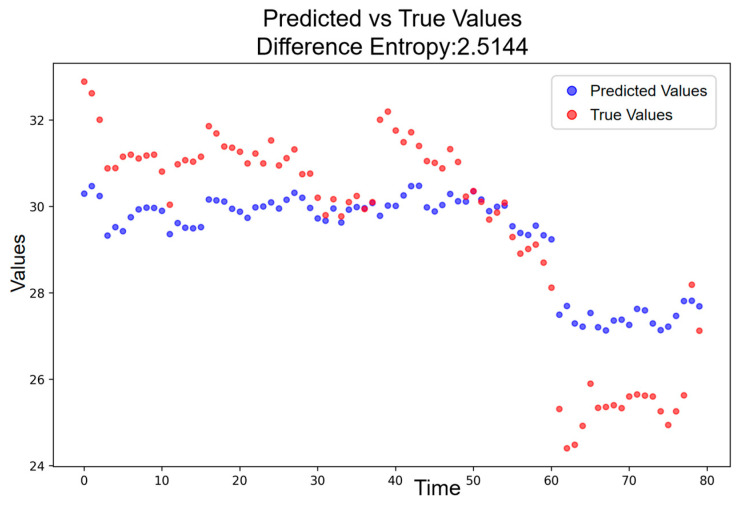
Predicted vs. Actual Price Trajectory during the Low- and High-Amplitude Periods.

To determine a quantitative threshold for differentiating low- and high-amplitude resonance, the entropy of the combined interval covering both phases is analyzed. As shown in Figure 12, the entropy for the merged period is H = 2.5144, yielding a phonetic-induced increase of ΔH = 0.8053. This intermediate ΔH value lies between the low-amplitude increase and the high-amplitude increase, providing a natural boundary for segmentation. Accordingly, ΔH thresholds can be used to classify resonance intensity.

The above results provide a clear and intuitive depiction of how the deviation entropy evolves across different resonance stages, revealing a pronounced contrast between the low- and high-amplitude periods. However, while these patterns are visually and descriptively distinct, relying solely on point estimates from individual intervals may lead to threshold choices that are sensitive to sampling variability. To establish a statistically grounded and robust criterion for amplitude segmentation, it is therefore necessary to incorporate uncertainty quantification into the threshold determination process.

To determine a quantitative threshold for differentiating low- and high-amplitude resonance, the entropy of the combined interval covering both phases is analyzed, and a bootstrap resampling procedure is employed to characterize the distribution and statistical uncertainty of the incremental entropy measure ΔH. Using the predicted and baseline price trajectories, the difference sequences are first computed, and their information entropy is measured. To account for statistical uncertainty in entropy estimation, a bootstrap procedure is applied, in which the difference sequences of both the predicted and baseline series are repeatedly resampled 1000 times, and the corresponding incremental entropy ΔH is computed for each resample, yielding an empirical distribution of entropy increments. The resulting bootstrap mean ΔH = 0.6395 with a 95% confidence interval of [0.0709, 1.0767] is used as a robust estimate of the phonetic-induced increase.

The intermediate ΔH value obtained for the combined period (ΔH = 0.8053) lies within the bootstrap confidence interval, supporting its use as a segmentation threshold. Accordingly, the ΔH thresholds derived from the bootstrap procedure can be used to classify resonance intensity: values near zero indicate the absence of widespread harmonic effects, intermediate increases correspond to low-amplitude resonance, and large ΔH values indicate high-amplitude resonance. The resulting classification is summarized in Table 2.

### 4.3. Empirical Study

#### 4.3.1. Sensitivity Analysis

To examine whether the entropy-based segmentation rule and the selected threshold are overly sensitive to specific correlation model specifications, we conduct a sensitivity analysis focusing on the DCC-GARCH component used to construct the dynamic correlation matrices. This exercise is motivated by the fact that the entropy increment ΔH is derived from prediction errors, which may vary with alternative parameterizations of the underlying volatility–correlation dynamics.

In the baseline setting, conditional correlations are estimated using a DCC-GARCH(1,1) model. To evaluate robustness, we re-estimate the correlation matrices using two alternative specifications, namely DCC-GARCH(1,2) and DCC-GARCH(2,1). These alternatives allow for different persistence structures in the conditional variance and correlation processes while remaining within the standard DCC-GARCH framework. For each specification, the resulting correlation matrices are embedded into the same DCC-RSR prediction model, ensuring that all downstream modeling choices are held constant.

**Figure 13:**
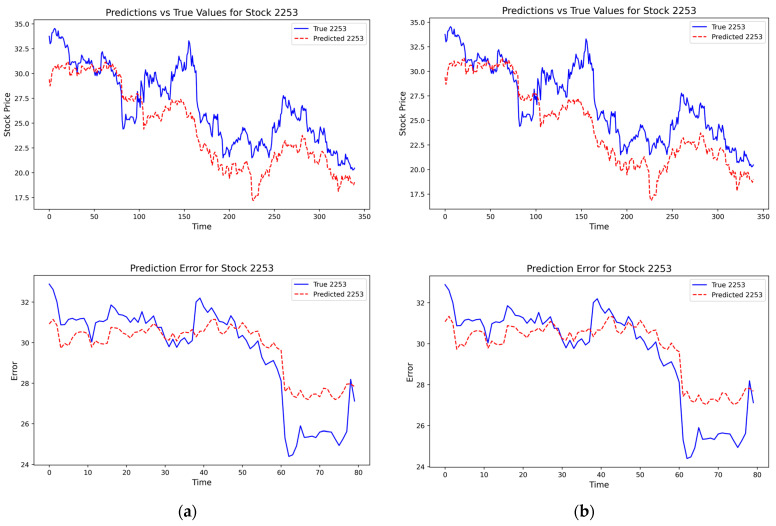
(**a**) Prediction results based on the DCC-GARCH(1,2) specification; (**b**) Prediction results based on the DCC-GARCH(2,1) specification.

Figure 13 compares the predicted price paths generated under the three DCC-GARCH specifications with the realized stock prices. The forecasts exhibit a high degree of similarity across specifications, particularly within the pan-homophonic event window, indicating that the predictive performance of the DCC-RSR model is not driven by a specific choice of DCC-GARCH orders.

To further assess the stability of the entropy-based segmentation rule, we extract the realized and predicted price series within the pan-homophonic event window for each specification and re-compute the entropy increment ΔH using a bootstrap resampling procedure. Specifically, we repeatedly resample the prediction-error series to obtain empirical distributions of ΔH, from which 95% confidence intervals are constructed. These bootstrap-based estimates are then compared with the original cutoff value ΔH = 0.6395 used in the baseline segmentation.

As shown in Table 3, the point estimates of ΔH remain close across alternative specifications, and the corresponding confidence intervals exhibit substantial overlap. Importantly, the baseline threshold lies well within the bootstrap confidence bands obtained under the alternative DCC-GARCH settings. This indicates that the entropy increment and the resulting regime segmentation are not materially altered by reasonable variations in the correlation model’s hyperparameters.

In addition to the sensitivity analysis on DCC-GARCH, we further examine the sensitivity of the segmentation results to the hyperparameters of the DCC-RSR prediction model, with a particular focus on the rolling-window length used in the LSTM-based ranking component. The rolling window determines the amount of historical information incorporated into each prediction and may therefore influence both forecasting behavior and the resulting entropy-based regime classification.

In the baseline specification, the DCC-RSR model is estimated using a rolling window of length 10. To assess robustness, we re-run the entire prediction procedure using two alternative window lengths, namely 5 and 20, while keeping all other model components unchanged.

**Figure 14:**
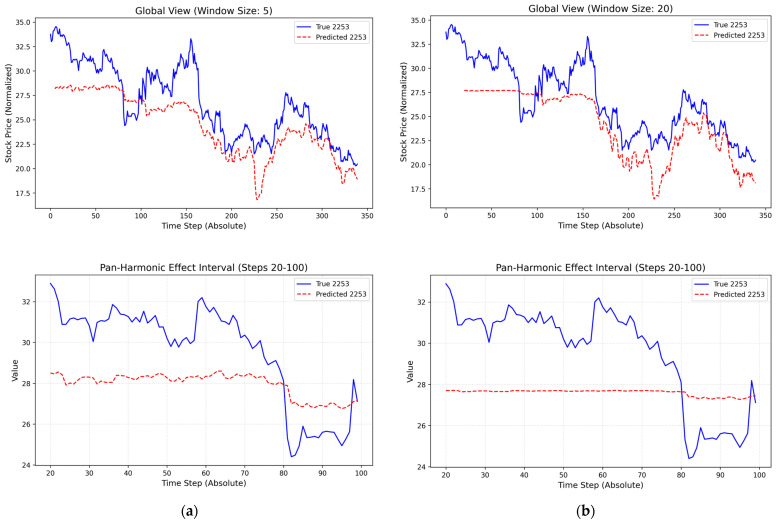
(**a**) Prediction results with 5 rolling-window lengths; (**b**) Prediction results with 20 rolling-window lengths.

Figure 14 compares the predicted and realized stock prices under rolling-window lengths of 5, 10 (baseline), and 20. Relative to the baseline, alternative window lengths lead to noticeable differences in the predicted price paths during the pan-homophonic event window. In particular, shorter and longer windows tend to underestimate periods of low-amplitude resonance and generate predictions with smaller price fluctuation ranges. Nevertheless, the overall temporal shape and directional movements of the predictions remain broadly similar across specifications.

These observations suggest that, while the DCC-RSR model captures the main dynamic pattern of the event window under different window lengths, its short-term sensitivity to resonance intensity is affected by the choice of rolling-window size, indicating room for further improvement in model robustness.

To evaluate whether such differences materially affect the entropy-based segmentation rule, we follow the same procedure as in the DCC-GARCH sensitivity analysis. Specifically, we extract the realized and predicted price series within the pan-homophonic event window for each window-length specification and re-compute the entropy increment ΔH using a bootstrap resampling procedure. This yields empirical distributions and 95% confidence intervals for ΔH, which are then compared with the baseline cutoff value ΔH = 0.6395.

As reported in Table 4, the point estimates of ΔH decrease as the rolling-window length departs from the baseline specification, and the corresponding confidence intervals widen and partially include zero. Two factors may contribute to this pattern. First, shorter or longer rolling windows alter the effective bias–variance trade-off of the LSTM-based predictor: shorter windows may insufficiently capture medium-term dynamics, while longer windows may oversmooth local fluctuations that are particularly relevant during resonance-driven event periods. Second, because the entropy increment is computed from prediction errors accumulated over a relatively short event window, changes in forecasting sensitivity can translate into amplified variability in ΔH.

In addition to the sensitivity analysis on the rolling-window lengths, we further evaluated whether the computed incremental entropy ΔH is sensitive to the choice of histogram binning used in the entropy calculation. To this end, three commonly employed binning rules were applied: Sturges, Freedman–Diaconis, and Scott. The resulting ΔH values are summarized in Table 5. It can be observed that the Sturges and Freedman–Diaconis rules both yield 8 bins with ΔH = 0.5979, whereas the Scott rule produces 6 bins with ΔH = 0.4454. Despite minor variations, all ΔH values remain within the previously reported 95% bootstrap confidence interval, indicating that the classification of low- and high-amplitude resonance is robust to the choice of binning method. This analysis confirms that the results of the entropy-based segmentation are not unduly influenced by the discretization of the deviation series.

#### 4.3.2. Placebo Test

To address concerns regarding potential false positives and to assess whether the identified pan-homophonic effect could arise spuriously from the modeling framework, a placebo test is conducted by shifting the event window to periods in which no pan-homophonic event occurred. Specifically, the original event window is moved forward by three months and six months, respectively. For each shifted window, the same empirical procedure is repeated: the DCC-GARCH-based correlation structure is used for prediction, the prediction error series is constructed, and the corresponding entropy difference ΔH is computed using a bootstrap approach identical to that applied in the original event analysis.

The results of the placebo test indicate that the entropy differences obtained from the shifted windows are substantially smaller than those observed during the original pan-homophonic event window. In particular, the bootstrap mean of ΔH equals 0.2821 for the three-month shifted window and 0.1497 for the six-month shifted window, both of which are far below the benchmark value of 0.6395 identified during the actual event period. A summary comparison between the original event window and the placebo windows is reported in Table 6.

Overall, the placebo evidence supports the interpretation that the observed pan-homophonic effect is unlikely to be driven by mechanical properties of the DCC-GARCH specification or the entropy-based metric, thereby reinforcing the causal relevance of the event window in generating the documented deviations.

To further assess the robustness of the identified pan-homophonic effect, a counterfactual test was conducted using a stock with similar characteristics to Chuandazhisheng 2253 but no plausible phonetic link. Huashengtiancheng 600410, identified based on sector membership and similarity rankings derived from the DCC-GARCH correlation matrix with a correlation of 0.5235 with 2253, was selected for this test. The full empirical pipeline including DCC-RSR-based prediction, construction of the prediction error series, and bootstrap-based ΔH computation was applied to 600410 over the Chuandazhisheng event window. The resulting entropy difference ΔH = −1.2456 fell clearly outside the threshold defining a pan-homophonic event. This counterfactual test thus reinforces the conclusion that the abnormal price deviations observed for Chuandazhisheng are specific to the pan-homophonic trigger rather than arising from general market or sectoral dynamics.

#### 4.3.3. Benchmark Comparison

To further assess whether the empirical results are driven by the specific choice of the DCC-GARCH-based correlation network, we extend the analysis by introducing alternative correlation estimators as benchmark models. The first benchmark replaces the DCC-GARCH estimator with a rolling Pearson correlation, computed over a fixed window, which allows correlations to vary over time but does not model conditional heteroskedasticity or dynamic covariance structures. The second benchmark adopts a constant conditional correlation GARCH (CCC-GARCH) model, which retains GARCH-based volatility modeling while imposing time-invariant conditional correlations across assets.

All benchmark correlation matrices are constructed using the same pre-event sample and are embedded into the identical LSTM prediction architecture. Predictive performance is evaluated using the mean squared error (MSE) between predicted and realized adjusted features of the focal stock during the event window. Under this unified setup, this benchmark design enables us to disentangle the incremental contribution of modeling dynamic conditional correlations from simpler dependence structures. Table 7 reports the comparative results.

Among all considered specifications, the DCC-GARCH model achieves the lowest MSE, indicating the best predictive performance. This justifies the use of the DCC-GARCH-based correlation matrix in the present study.

To further assess the robustness of the predictive framework, the DCC-RSR model was benchmarked against three alternative models, Random Walk (RW), ARIMA, and LSTM, over the same non-event periods. The mean squared errors (MSEs) for each model are presented in Table 8.

The results indicate that the DCC-RSR model achieves the lowest MSE, outperforming both conventional benchmarks, Random Walk and ARIMA, and the LSTM model without correlation network input. This provides evidence that the model’s predictions during non-event periods are highly accurate, ensuring that the abnormal components identified during the event windows are unlikely to be artifacts of general prediction errors or regime shifts. Consequently, the observed deviations can be interpreted as genuine anomalies, supporting the empirical validity of the findings.

#### 4.3.4. Confounder-Adjusted OLS Regression

To examine whether the discrepancy between realized prices and model-implied benchmark predictions can be attributed solely to the pan-homophonic effect, or whether it is partially driven by concurrent confounding factors, we conduct a confounder-adjusted ordinary least squares (OLS) regression. The dependent variable captures the abnormal deviation between the observed stock price and the corresponding predicted value generated by the benchmark model.

Formally, we estimate the following regression specification:

(10) $$Y_{i,t}=\alpha +\beta_{1}Event_{i,t}+\beta_{2}Market_{t}+\beta_{3}Turnover_{i,t}+\varepsilon_{i,t},$$

where $Y_{i,t}$ denotes the price deviation for stock i at time t. $Event_{i,t}$ is a pan-homophonic event dummy, $Market_{t}$ represents the market return, and $Turnover_{i,t}$ captures stock-level liquidity conditions.

The choice of control variables reflects the institutional context of the focal stock. As Chuandazhisheng is listed on the Shenzhen exchange, the market control variable is constructed as the daily return of the Shenzhen Composite Index. Liquidity conditions are proxied by the stock’s turnover rate, which is commonly used to capture trading intensity and retail participation.

The pan-homophonic effect is operationalized as a binary indicator that equals one only during the High-Amplitude Periods, and zero otherwise. This design reflects our theoretical argument that pan-homophonic resonance is economically meaningful only during episodes of strong phonetic salience. In contrast, Low-Amplitude Periods are characterized by relatively muted price movements and are therefore not expected to generate statistically detectable effects. All non-event periods are also coded as zero.

Based on the observed price dynamics during the High-Amplitude Periods—namely, a short-lived price surge followed by a prolonged decline lasting over one month—we hypothesize a negative association between the pan-homophonic event indicator and the realized prediction error. Intuitively, heightened phonetic resonance may induce temporary price inflation relative to model-implied benchmarks, followed by systematic correction, resulting in a negative average deviation during the event window. By contrast, we do not expect market-wide movements to explain the abnormal deviations once the benchmark model has already absorbed common market information.

The estimation results are reported in Table 9.

The coefficient on $Event_{i,t}$ is negative and highly statistically significant. This finding indicates that, conditional on market returns and liquidity controls, the occurrence of a high-amplitude pan-homophonic event is associated with a substantial downward deviation of realized prices relative to their predicted benchmarks. Economically, this pattern is consistent with a transient price overshoot driven by non-fundamental phonetic cues, followed by a correction phase in which prices fall below the model-implied trajectory.

Consistent with our prior conjecture, the market return variable is statistically insignificant, suggesting that the observed deviations are not driven by broad market upswings or downturns. This supports the interpretation that the price dynamics of Chuandazhisheng during the event window are not a mechanical reflection of overall market conditions.

In contrast, the turnover rate enters the regression with a positive and highly significant coefficient. We interpret this result as evidence that liquidity measures partly internalize the pan-homophonic mechanism itself. Specifically, the emergence of pan-homophonic resonance may stimulate non-rational trading demand and intensified participation by retail investors, thereby mechanically increasing turnover. As a result, liquidity variables are not orthogonal to the pan-homophonic effect, which explains their strong statistical significance even after controlling for the event indicator.

## 5. Discussion

To further explore whether there have been changes in the current response of the Chinese stock market to the pan-homophonic effect, we took Donald Trump’s election as President of the United States on 6 November 2024, as an example and input it into the RSR model for further verification. After the news of Trump’s election was announced, we observed that the stock price trend of Chandazhisheng was different from that under the pan-homophonic effect in 2016.

Following the same empirical procedure as in the 2016 analysis, we additionally quantify the pan-homophonic effect during the 2024 episode using the entropy-based deviation measure. Specifically, the incremental entropy is computed from the prediction errors under an identical bootstrap protocol, allowing for a direct and consistent comparison across periods.

**Figure 15:**
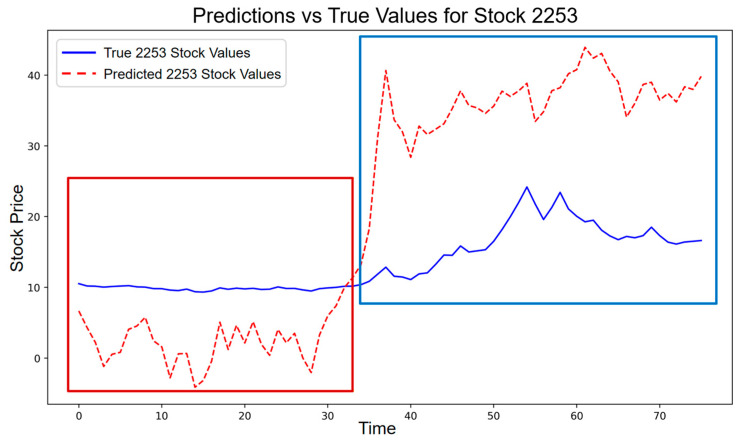
Stock price trend of the pan-homophonic effect in 2024 (red box: low-amplitude resonance; blue box: high-amplitude resonance).

Specifically, during the preheating period of the US presidential election, the stock price of this stock showed a slight deviation from the predicted value, which was consistent with the stock price performance in 2016 and conformed to the characteristics of the low-amplitude resonance period. Consistent with this visual pattern, the entropy-based measure during the low-amplitude phase yields an average incremental entropy of ΔH = 0.2359, which falls within the low-amplitude resonance interval according to the validated segmentation rule. Before and after the news was announced, there was no high-amplitude resonance phenomenon of sharp increase in a short period of time as in 2016. The stock price even fluctuated below the predicted level and tended to be stable, as shown in Figure 15.

In line with this observation, the entropy deviation measured during the high-amplitude phase is ΔH = −0.9328, indicating that no pan-homophonic resonance is detected in this period. At the aggregate level, the overall entropy deviation for the entire 2024 pan-homophonic window equals ΔH = −1.0804, suggesting that the pan-homophonic effect is statistically insignificant in 2024.

The fact that the actual stock price tends to be stable under the pan-homophonic effect indicates that the Chinese stock market has become relatively mature. With the continuous improvement of the regulatory system of the Chinese stock market, the increasing transparency of the information disclosure system, and the general improvement of investors’ education level, the ability of market participants to screen and judge information has significantly increased.

Nowadays, in the face of the pan-homophonic effect, investors are more inclined to conduct in-depth research, analyzing factors such as a company’s fundamentals, profitability, and industry prospects, rather than blindly following trends based solely on concepts or slogans. They recognize that short-term market enthusiasm is unlikely to sustain stock prices, and that long-term value is the key determinant of a stock’s performance. Therefore, even when similar hype hotspots occasionally appear in the market, they are unlikely to trigger large-scale irrational investment behavior, and the market’s self-regulating ability has been effectively enhanced.

Theoretically, the attention spillover theory provides a powerful framework for explaining the differences in the performance of the pan-homophonic effect at different time points. The entropy-based evidence further supports this interpretation by showing that, unlike in 2016, the 2024 episode fails to generate a statistically meaningful high-amplitude resonance. Specifically, when the pan-homophonic effect occurred for the first time, since investors were relatively unfamiliar with this kind of market anomaly triggered by the similarity in pronunciation between stock codes or names and specific events or people, they often paid extremely high attention. This high-level market focus led to a sharp increase in the stock prices of related stocks in a short period, forming a significant pan-homophonic effect. During this process, some investors made substantial profits by successfully seizing this market opportunity. As a result, due to over-confidence, their attention spilled over to other stocks with similar characteristics or potential relevance, further fueling market volatility.

However, when the pan-homophonic effect occurred for the second time, investors’ behavior patterns changed significantly. Influenced by past positive feedback trading experiences and the continuous effect of the over-confidence factor, although investors still reacted to the new pan-homophonic event, the intensity of their reaction was much weaker. This attenuation is quantitatively reflected in the markedly lower and even negative entropy deviations observed in 2024, which suggests that investors’ attention had become more dispersed and less concentrated on a single pan-homophonic cue.

Therefore, we refer to this phenomenon as the pan-homophonic effect spillover, which reveals that when a similar event occurs again, the dispersion of investor attention and the increased emphasis on fundamentals substantially weaken the price impact of pan-homophonic cues, resulting in a much lower level of stock price surges compared to the first occurrence.

## 6. Conclusions

This research is based on the theoretical frameworks of the Efficient Market Hypothesis and Behavioral Finance. By applying complex network simulation techniques, it deeply analyzes the evolutionary mechanism of the impact of the pan-homophonic effect on stock prices and explores the complex phenomena of stock price anomalies under the action of the pan-homophonic effect. Meanwhile, this research innovatively proposes the language amplitude division hypothesis for hot events, which is based on information entropy, to deepen the understanding of the driving factors behind stock price fluctuations. Through an empirical case of the abnormal stock price fluctuations of Chuandazhisheng during the US presidential election, this research uses the DCC-GARCH model to analyze in detail the impact of the pan-homophonic effect on the dynamic relationships among listed companies in the “software and information technology services industry”. On this basis, this research adopts the Stock Relationship Ranking Prediction (RSR) Model and, under the theoretical assumption of the Efficient Market Hypothesis, simulates the stock price trend of Chuandazhisheng under normal market conditions. Through comparative analysis, this research reveals the significant differences in the stock price trends of Chuandazhisheng between the situation of the Efficient Market Hypothesis and the market anomalies triggered by the pan-homophonic effect, thus further enriching the theoretical connotation of market behavior complexity and deepening the understanding of market driving factors. Through in-depth analysis of the market anomalies of Chuandazhisheng and based on the known high correlation between hot events and stock market anomalies, our further comparative research findings are as follows: First, under the pan-homophonic effect, stock prices evolve from the low-amplitude resonance period to the high-amplitude resonance period. Second, during the low-amplitude resonance period, the stock price exhibits a moderate increase relative to the baseline, corresponding to a phonetic-induced information entropy that is less than or equal to 0.8053. During the high-amplitude resonance period, the stock price shows a pronounced deviation, associated with a phonetic-induced information entropy that is greater than 0.8053. Third, in the short-term trend, stock prices often exhibit unsustainable and temporary characteristics. The sharp increase phenomenon is limited to the day of the hot event or one to two days thereafter. In the long-term trend, the stock price quickly drops back below the normal level and lasts for about three weeks. Fourth, during the second pan-homophonic event, the degree of the stock price surge is much lower than that of the first time, and the effect of the pan-homophonic effect is weakened, which is called the pan-homophonic effect spillover. These findings collectively demonstrate that the stock price evolution indeed follows the transition from low-amplitude disturbances to high-amplitude surges and subsequent rapid corrections, thereby providing evidence consistent with Hypothesis 1 and confirming the proposed dynamic resonance mechanism induced by the Pan-Homophonic Effect.

The research results of this paper reveal the irrational state of investors in speculative activities, mainly reflected in aspects such as arbitrage psychology and herd mentality, and this phenomenon is particularly significant in the market dynamics of the transition from the low-amplitude resonance period to the high-amplitude resonance period. Specifically, non-substantive related events in the market, such as homophonic stems, first slightly disturb the stock price during the low-amplitude resonance period. Although these events have no direct relation to the stock fundamentals, they can still trigger a certain market reaction in the short term. Entering the high-amplitude resonance period, the stock price anomalies triggered by similar events are more intense, the stock price volatility significantly increases, accompanied by a large value deviation. At this stage, the irrational association between high-amplitude events and stock prices leads to sharp rises and falls in stock prices. If investors blindly follow the trend, they may suffer significant losses. Therefore, for high-amplitude events, investors need to be highly vigilant, rationally evaluate their actual impact, and avoid being misled by short-term market noise. In contrast, although low-amplitude events in the low-amplitude resonance period can also cause stock price anomalies, the resulting value deviation is relatively small, and they have the characteristics of concealment and persistence. The impact of such events on stock prices is relatively gentle and long-term, providing investors with a relatively ample time window for calm analysis and decision-making. Secondly, it is particularly important that investors should avoid blindly investing in stocks related to the second pan-homophonic event due to the stock price surges caused by past pan-homophonic events. Due to the existence of the pan-homophonic effect spillover, the degree of the stock price surge during the second event is much lower than that of the first time, and its effect is greatly weakened. Overall, when facing market fluctuations, investors should uphold a long-term investment perspective, rationally analyze the fundamentals of enterprises, industry prospects, and market trends. Especially during the transformation from the low-amplitude resonance period to the high-amplitude resonance period, investors need to be more cautious about market anomalies, especially when high-amplitude events occur, to avoid being confused by short-term fluctuations and ensure the long-term and stable appreciation of assets.

## Figures and Tables

**Table 1:** Methodological Comparison between Original RSR and the Proposed DCC-RSR in the Context of Homophonic Spillover Detection.

Component	Original RSR [23] ^1^	Proposed DCC-RSR
Task-Formulation	Learning-to-rank for stock return prediction	Same
Temporal Embedding	$\mathrm{LSTM}\;\mathrm{applied}\;\mathrm{to}\;\mathrm{input}\;\mathrm{sequence}\;x_{t}$ $\;\mathrm{to}\;\mathrm{obtain}\;\mathrm{temporal}\;\mathrm{embedding}\;h_{t}$	Same
Relation-Construction	Static multi-relational tensor $A\in {\mathbb{R}}^{N\times N\times K}$, based on sector–industry relations and Wiki company-based relations among stocks	$\mathrm{Dynamic}\;\mathrm{correlation}\;\mathrm{matrix}\;C_{t}\in {\mathbb{R}}^{N\times N}$, estimated by DCC-GARCH
Graph Properties	Static, discrete, predefined; relation types are fixed	Dynamic, continuous, updated daily with return data
Graph-Convolution	Temporal Graph Convolution (TGC) over A	TGC over dynamically updated DCC-based graphs
Relation-Weight Function	$g(e^{i},e^{j})={\left(e^{i}\right)}^{T}e^{j}$ or MLP over embeddings	Inner product over dynamic embeddings
Prediction-Output	Next-day return ranking score	Same
Application Focus	Integrates sequential and structural learning under fixed relational assumptions	Detection of homophonic-driven price deviations via counterfactual return ranking

^1^ The original Relational Stock Ranking (RSR) model was introduced by Feng et al. [23] to jointly learn temporal and structural patterns in stock return ranking. The DCC-RSR model proposed in this study extends this framework by integrating dynamic inter-stock correlations estimated via the DCC-GARCH [21,22] model. This modification enables the identification of return deviations arising from homophonic spillover effects, where stock names or ticker symbols phonetically resemble exogenous event terms.

**Table 2:** Language Amplitude Division by the Incremental Entropy.

Amplitude Division	ΔH (Entropy Increase over Baseline)
No widespread harmonic effect	≤0
Low-Amplitude resonance period	>0 and ≤0.6395
High-Amplitude resonance period	>0.6395

**Table 3:** Bootstrap estimates of entropy increments under alternative DCC-GARCH specifications.

DCC-GARCH Specification	ΔH	95% Confidence Interval
GARCH(1,1)	0.6395	[0.0709, 1.0767]
GARCH(1,2)	0.7244	[0.1085, 1.1635]
GARCH(2,1)	0.7762	[0.2068, 1.2072]

**Table 4:** Bootstrap estimates of entropy increments under alternative rolling-window lengths.

Rolling-Window Length	ΔH	95% Confidence Interval
10	0.6395	[0.0709, 1.0767]
5	0.3703	[−0.2274, 0.7390]
20	0.1481	[−0.4661, 0.5160]

**Table 5:** Sensitivity of ΔH to binning method.

Binning Rule	Number of Bins	ΔH
Sturges	8	0.5979
Freedman–Diaconis	8	0.5979
Scott	6	0.4454

**Table 6:** Placebo test based on shifted event windows.

Event Window Specification	ΔH (Bootstrap Mean)
Original pan-homophonic event window	0.6395
Placebo window (+3 months)	0.2821
Placebo window (+6 months)	0.1497

**Table 7:** Predictive performance under alternative correlation estimators.

Correlation Estimator	Heteroskedasticity Modeling	Dynamic Correlation	MSE
Rolling Pearson Correlation	No	Yes	2.5199
CCC-GARCH	Yes	No	5.3076
DCC-GARCH	Yes	Yes	1.1618

**Table 8:** MSE for alternative predictive models (non-event period).

Model	MSE
Random Walk	4.0378
ARIMA	4.1344
LSTM	12.1209
DCC-RSR	1.1618

**Table 9:** Confounder-Adjusted OLS Regression Results.

Variable	Coef.	Std. Err.	t-Stat	*p* > |t|	95% CI Lower	95% CI Upper
α	2.3010	0.100	22.975	0.000	2.104	2.498
Event	−3.1212	0.186	−16.742	0.000	−3.488	−2.755
Market	8.5737	6.923	1.238	0.216	−5.044	22.191
Turnover	31.4647	2.636	11.935	0.000	26.279	36.650

## Data Availability

Data is available from the corresponding author upon reasonable request.

## Abbreviations

The following abbreviations are used in this manuscript:

EMH	Efficient Market Hypothesis
RSR	Relational Stock Ranking
DCC	Dynamic Conditional Correlation
GARCH	Generalized Autoregressive Conditional Heteroskedasticity
LSTM	Long Short-Term Memory
TGC	Temporal Graph Convolution
MSE	Mean Squared Error
